# Balling Behavior of Selective Laser Melting (SLM) Magnesium Alloy

**DOI:** 10.3390/ma13163632

**Published:** 2020-08-17

**Authors:** Shuai Liu, Hanjie Guo

**Affiliations:** 1 School of Metallurgical and Ecological Engineering, University of Science and Technology Beijing, Beijing 100083, China; lsls1226@163.com; 2 Beijing Key Laboratory of Special Melting and Preparation of High-End Metal Materials, University of Science and Technology Beijing, Beijing 100083, China

**Keywords:** selective laser melting, magnesium and alloys, balling behavior, thermodynamics and kinetics

## Abstract

Macroscopic surface morphology and balling mechanism of AZ61 magnesium alloy prepared by Selective laser melting (SLM) have been investigated. This article studied and analyzed the surface morphology and balling phenomenon of Mg in the laser processing from the aspects of Mg inherent metal properties and laser processing. In terms of laser processing, the results show that, in the direction of increasing scanning speed, the energy density decreases, and the phenomenon of balling and porosity on the surface of the magnesium alloy is serious. When the energy density is 133.9–187.5 J/mm^3^, balling particles are significantly reduced. It can be seen from the low-magnification SEM image that, even at a scanning speed of 250 mm/s (E_v_ is 187.5 J/mm^3^), there are still a few small-sized balling particles on the surface. Therefore, in terms of inherent metal properties, the wettability, capillary instability, thermodynamic, and kinetic analysis of the balling behavior of Mg and other metal (Al, Fe, Cu, Ni, Ti) droplets in the SLM process has been carried out, and the dynamic model of magnesium droplet spreading/solidification was established basic on the result of experiment and metal inherent properties. The results show that SLMed magnesium alloy is a competitive process of melt diffusion and solidification. The final result depends on the intrinsic properties of the magnesium alloy and the applied laser processing parameters. The spreading process of Mg melt is very fast. Although the solidification time of Mg melts changes slowly with the increase of metal droplet temperature, the spreading speed is still very fast due to the low melt density, so the balling phenomenon of SLMed Mg can be controlled to a certain extent. Theoretically calculated, the solidification time of Mg melt droplet is longer than the wetting time at 1173 K (900 °C), so the spreading process is dominant, which can minimize the balling and realize the densification of SLMed Mg. The dynamic spreading of molten pool, the analysis of wetting and solidification process, and the establishment of SLM balling model can provide reference for the design of the SLM forming parameters of Mg and other different metals.

## 1. Introduction

Magnesium and its alloys meet the needs of high efficiency and sustainable development. Magnesium alloy, as one of the advanced metal materials, is widely used in aerospace, automobile manufacturing, biomedicine, and other industries due to its advantages of high specific strength and light weight [1,2,3]. Additionally, there is the potential and possibility to replace aluminum and steel in many structural applications [4]. However, these advantages of magnesium and its alloys still have many difficulties to overcome in industrial applications. For instance, the poor creep resistance, tensile properties (strength and ductility), and corrosion resistance of magnesium alloys. With the continuous expansion of the application field of advanced magnesium alloy materials, the complex structure, efficient and environmentally friendly production requirements have become the focus of the development of magnesium alloys, so new magnesium alloy preparation technologies have emerged at the historic moment.

Selective laser melting (SLM) evolved from selective laser sintering (SLS). It is a layered manufacturing technology that is characterized by a fast solidification rate and it can directly manufacture parts with complex geometric shapes [5,6,7]. The powder bonding in the SLM process does not require binding materials, so downstream processes are eliminated. Therefore, the current SLM technology has improved product quality, processing time, and manufacturing reliability when compared to other processes. On the one hand, SLM can completely melt powder materials and produce dense and close-to-net-shape parts without post-processing and removing parts and supports from the substrate. This is a process with special capabilities to directly produce metal parts with complex geometries, enabling researchers to study parts used in the aerospace and automotive industries, such as cutting tools, conformal cooling channels, and Sidewinder missile guidance section shell for lightweight structures [8,9]. On the other hand, according to properties, such as high strength, high ductility, and good biocompatibility, SLMed materials have many applications. Because the current SLM process is expensive when compared with most traditional manufacturing methods, it is proposed to be applicable to high value-added industries, such as the pharmaceutical industry [10,11]. For example, the dental crowns produced by SLM [12]. It was also reported that, as compared with stainless steel and titanium alloys, magnesium alloys are more compatible with the mechanical properties of human bones, so, based on the advantages of the SLM process, SLMed magnesium alloys can directly produce human bone implant materials [13], such as an additively manufactured magnesium alloy human jaw [14].

However, the metal parts prepared by SLM suffer from the problem of balling. Laser-induced balling is an important factor that affects the surface quality of SLMed parts and the densification of samples. Balling is some spherical agglomerates on the surface of SLMed components [15]. Balling often occurs in SLMed magnesium alloys. Ng C. C. et al. [16] believed that the melting track of SLMed pure Mg is composed of a series of discontinuous spherical particle, which are gathered together to form a single track. The formation of these spherical particles is affected by surface tension, which prevents the melt from spreading uniformly, thus forming spheres, which are usually called "balling". In SLMed AZ91, when the laser energy is lower than 77 J/mm^3^, the manufactured samples are loose due to “balling effect” and incomplete powder [17]. As the energy density increases, the balling phenomenon improves. However, Liu et al. [18] reported that SLMed Mg-Ca alloy exhibited balling at both low and high energy input. The “balling effect” on the surface of magnesium alloys is serious, with E below 750 J/mm^3^. The best energy density for SLMed Mg-Ca is between 875 J/mm^3^ and 1000 J/mm^3^. At this time, the sample shows that there are fewer balling particals and the surface is smooth. The balling phenomenon is serious when the energy density is lower than the optimal range. Above the optimal range, the pore size of the sample becomes smaller, but the surface is rough.

Thence, the balling effect in SLMed Mg is a problem that is intractable and needs to be discussed in depth. Gu et al. [19] investigated the mechanism of balling. It is believed that the phenomenon of balling is closely related to the thermodynamic and dynamic characteristics of laser sintering and it is controlled by a series of process parameters. According to different formation conditions, balling can be divided into three categories: the first kind of balling, shrink balling, and self-balling. The research of Li et al. [20] showed that there are two types of balling of SLM: one has a large diameter (500 μm) and its shape is mainly ellipsoid; the other has a small diameter (10 μm) and it is mainly spherical. The formation of the first type of balling particles is due to poor wettability, which leads to more serious balling of SLM; the second type of balling particles does not significantly damage the performance of the SLM sample. It can be seen that most of the current researches control balling particles from the perspective of SLM processing, such as adjusting process parameters. It is believed that the adjustment of process parameters will affect the flow stability and surface tension, which, in turn, affects the phenomenon of balling. However, in addition to controlling balling particles from the perspective of SLM process, the study of the inherent characteristics of materials is very worthwhile and necessary for the balling control in the SLM process. The balling phenomenon is also closely related to the material properties (melting point, density, thermal conductivity, heat capacity). At present, there are few studies on controlling the balling of SLMed magnesium alloys, especially the lack of research on the coupling of magnesium alloy material properties with processing parameters. 

As for SLMed magnesium alloys, scholars have studied the influence of laser power on sample forming and quality [21,22,23]. For SLMed AZ61 magnesium alloy [23], studies have shown that with the increase of laser power, the relative density of the sample increases, and the microstructure presents equiaxed crystals, which improves the material’s degradation resistance and microhardness. Too high laser power will lead to a decrease in the amount of solid solution of aluminum in the Mg matrix and the coarsening of the equiaxed grains, which results in a decrease in relative density and microhardness. In view of the lack of effects of scanning speed and hatch spacing on the forming of SLMed AZ61 magnesium alloy, the previous work focused on the two process parameters of scanning speed (1800–250 mm/s) and hatch spacing (0.06–0.01 mm) to study the morphology and performance of the SLMed AZ61 magnesium alloy samples. The single-layer experimental results in the previous work show that the hatch spacing has a greater influence on the intra-layer bonding. The hatch spacing mainly affects the combination of two adjacent tracks, thereby affecting the relative density of the sample. As the hatch spacing increases, the gap between adjacent tracks increases, but the morphological characteristics of the sample, especially the balling particles, more obviously change with the scanning speeds. Therefore, this work will mainly focus on the influence of scanning speed in order to further study the balling of SLMed magnesium alloy. In addition, the quality of SLMed magnesium alloy samples is better when the hatch spacing is 0.06–0.08 mm. Additionally, when the hatch spacing is 0.08 mm, some new phenomena are found in the variation of the surface morphology with the scanning speeds. The processing parameters can control the balling to a certain extent, but it has limitations. Hence, the control of balling requires further in-depth study. 

In this work, we present new findings on the variation of scanning speeds when the hatch spacing is 0.08 mm and conduct in-depth research on the balling mechanism from the perspective of material intrinsic properties. This work studied and analyzed the surface morphology and balling phenomenon of Mg in the laser processing process from the aspects of Mg inherent metal properties and laser processing in order to further clarify the balling phenomenon of Mg in the laser processing process. Additionally, establish a model of balling and seek the best way to control the balling of SLMed Mg.

## 2. Material and Methods 

### 2.1. Materials

**Figure 1:**
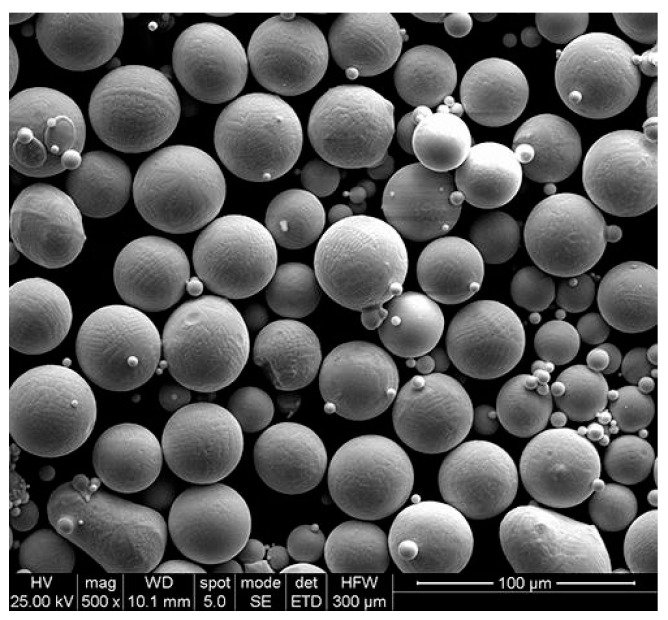
AZ61 magnesium alloy powders [24].

The gas atomized spherical AZ61 magnesium alloy powder is a powder for experiment (provided by Sichuan Kehui Industrial Co., Ltd., Chengdu, China). The average particle size of the powder is 48 μm, and the particle size distribution is between 30 μm to 70 μm (Figure 1), and it is measured by the laser particle size analyzer LMS-30. Table 1 shows the chemical composition of the experimental magnesium alloy powder. Inductively coupled plasma emission spectroscopy (ICP IRIS Intrepid II, Waltham, MA, USA) and oxygen nitrogen hydrogen analyzer (TCH-600, Michigan, MI, USA) determine the composition.

### 2.2. Experimental System and Processing

The experiments were carried out on an SLM machine FORWEDO LM-120 (Harbin, China). The laser of the SLM equipment is an IPG YLR-500 fiber laser (the laser type is a continuous wave), the maximum output power is 2 kW, and the wavelength is 1064 nm, and the machine includes an automatic powder feeding device and an atmosphere protection system. The experiment was carried out under the protection of argon, the content of O_2_ and H_2_O was below 0.01 vol.%, and the molding cavity size is 120 × 120 × 160 mm^3^. A rolled AZ31 (nominal composition Mg-3wt.%Al-1wt.%Zn) is used as the substrate and the size of the substrate is 120 × 120 × 12 mm^3^. More details regarding the SLM apparatus can be found in Refs. [24,25]. No preheating was used throughout the experiment.

**Figure 2:**
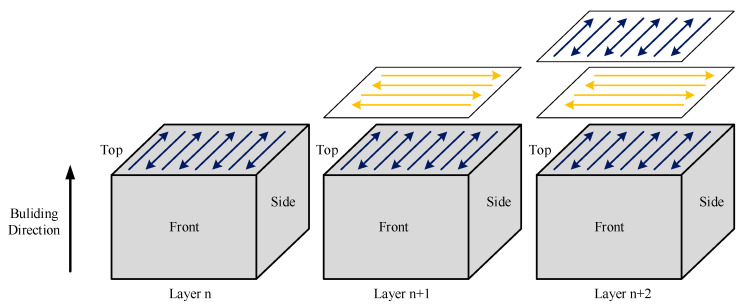
Illustration of the selective laser melting (SLM) process.

The experimental process is shown in Figure 2, the powder is spread on the substrate, the laser melts the powder according to the zigzag scanning strategy, and rotates 90° in the next layer. Block samples are completed after printing layer by layer. Table 2 shows the process parameters used in the experiment.

### 2.3. Microstructural Characterization

The size of SLMed AZ61 magnesium alloy sample is 10 × 10 × 7 mm^3^. The scanning speed ranges from 250 mm/s to 950 mm/s with an interval of 50 mm/s. The hatch spacing was set to 0.08 mm according to the previous work [24]. In the SLM process, the layer thickness not only affects the construction rate, but also directly affects the characteristics of the molten pool, such as mass transfer, heat transfer, and cooling rate. Studies have shown that the layer thickness will affect the macroscopic morphology, microstructure, and mechanical properties of SLMed samples [26,27]. A thicker layer thickness will result in the appearance of residual micropores in the SLMed sample and affect the relative density. Additionally, the grain size increases with the increase of the layer thickness, thus affecting the performance of the sample [27]. Accordingly, the thinner the layer thickness, the more fully the powder is heated, the faster the cooling rate of the single layer, and the better the density and dimensional accuracy of the sample [28]. However, if the layer thickness is too low, then the construction rate will be slow, and it will cause uneven spreading due to the mismatch with the powder particle size. Therefore, when considering the particle size of the powder, the uniformity of spreading powder, the construction rate and the molding quality, the layer thickness is set to a moderate 40 μm. Additionally, the laser power is set to 150 W in reference to the previous work and related research of SLMed magnesium alloy [17,23,24]. Other processing parameters remained unchanged. A scanning electron microscope (SEM; JSM-6701F, Tokyo, Japan) was used to observe the microstructure of the sample, and the accelerating voltage was 20 kV. The relative density was measured by the Archimedes method.

## 3. Results and Discussion

### 3.1. Study of Scanning Speed and Energy Density

**Figure 3:**
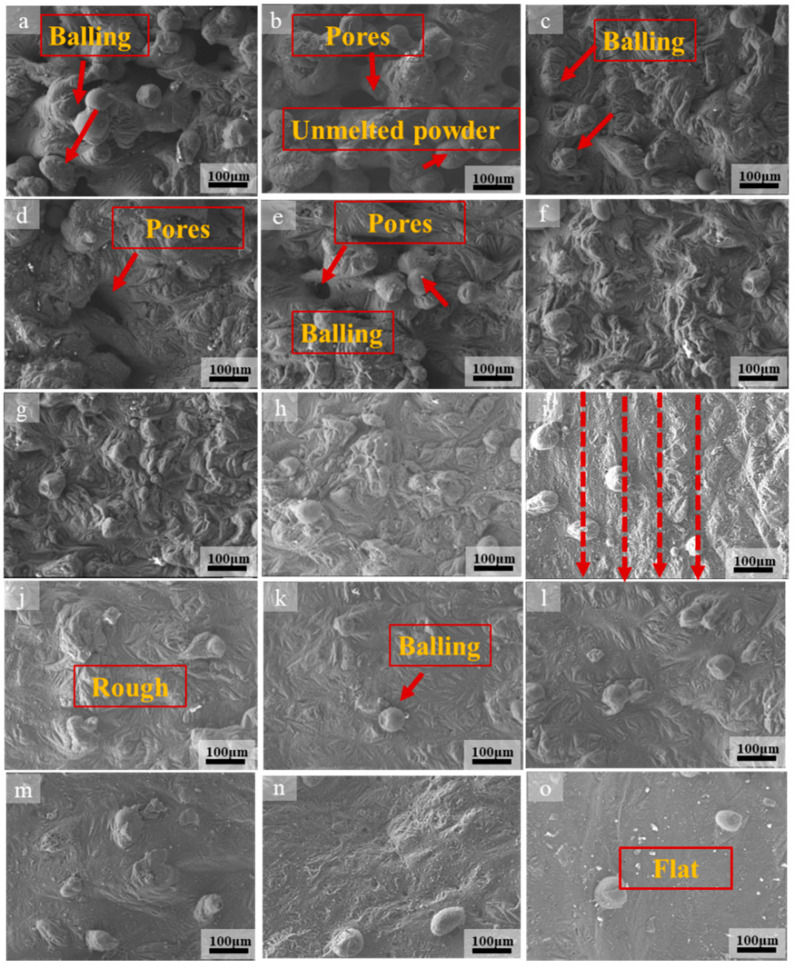
High-magnification scanning electron microscope (SEM) image surface of morphology of SLMed AZ61 magnesium alloy under different scanning speeds: (**a**) 950 mm/s, (**b**) 900 mm/s, (**c**) 850 mm/s, (**d**) 800 mm/s, (**e**) 750 mm/s, (**f**) 700 mm/s, (**g**) 650 mm/s, (**h**) 600 mm/s, (**i**) 550 mm/s, (**j**) 500 mm/s, (**k**) 450 mm/s, (**l**) 400 mm/s, (**m**) 350 mm/s, (**n**) 300 mm/s, and (**o**) 250 mm/s.

**Figure 4:**
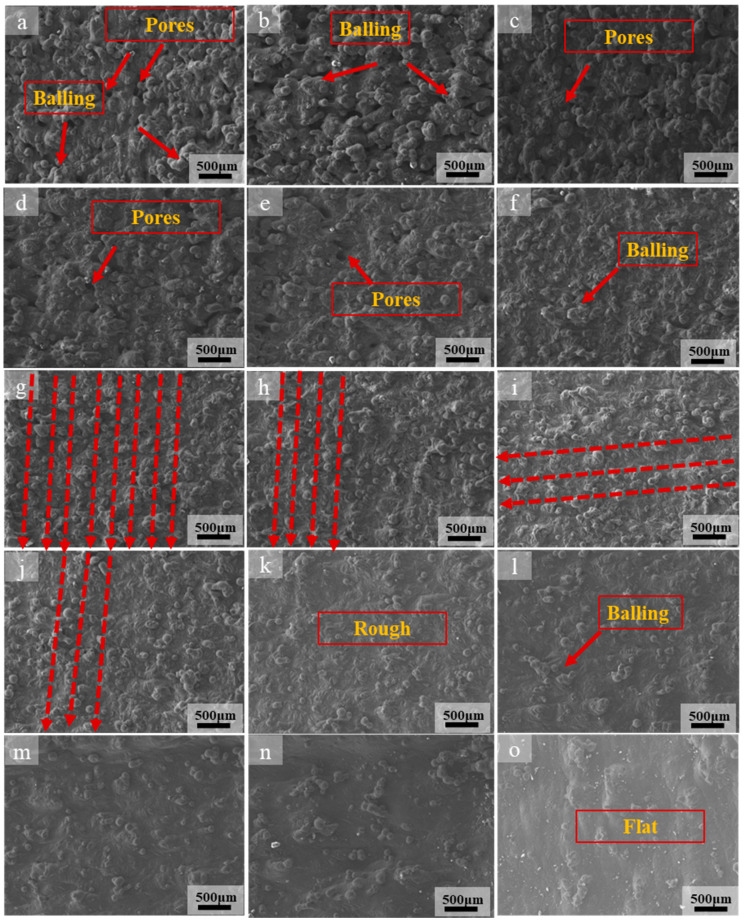
Low-magnification SEM image of surface morphology of SLMed AZ61 magnesium alloy under different scanning speeds: (**a**) 950 mm/s, (**b**) 900 mm/s, (**c**) 850 mm/s, (**d**) 800 mm/s, (**e**) 750 mm/s, (**f**) 700 mm/s, (**g**) 650 mm/s, (**h**) 600 mm/s, (**i**) 550 mm/s, (**j**) 500 mm/s, (**k**) 450 mm/s, (**l**) 400 mm/s, (**m**) 350 mm/s, (**n**) 300 mm/s, (**o**) 250 mm/s.

Figure 3 shows the high-magnification SEM image of the surface morphology of the SLMed magnesium alloy sample and Figure 4 shows the corresponding low-magnification SEM image.

It can be seen from Figure 3 that the surface morphology of SLMedAZ61 magnesium alloy is related to the applied processing parameters. The energy density is introduced as a standard for quantitative analysis in order to facilitate the analysis. Some research work has also verified that energy density is related to the quality of SLM samples [10,11]. Energy density E_v_ is related to processing parameters, and it can be represent as

(1) $$E_{v}=\frac{P}{\upsilon \cdot H\cdot T}\left({J/\mathrm{mm}}^{3}\right)$$

where *P* represents the laser power (W), *H* is the hatch spacing (mm), υ represents the scanning speed (mm/s), and *T* is the layer thickness (mm).

**Figure 5:**
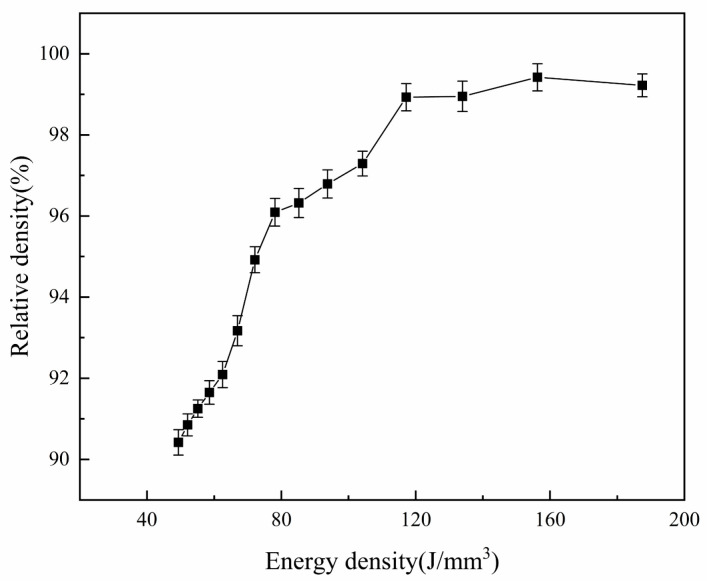
The relationship between energy density and relative density of SLMed magnesium alloy.

As the scanning speed changes, some typical surface topography appears. When applying a higher scanning speed (950, 900 mm/s), the E_v_ is 49.3–52.1 J/mm^3^. The pores, unmelted powders, and agglomerated balling on the surface are more serious, and pores and balling particles with larger sizes appear on the surface (Figure 3a,b and Figure 4a,b red arrow). The balling particles and pores on the surface of the previous layer not only affect the intra-layer binding, but also affect the inter-layer binding, thus affecting the relative density of the sample. Figure 5 shows the relative density under the corresponding energy input. As can be seen from the Figure 3a,b and Figure 4a,b, the diameter of some spherical particles is large (about 200 μm), and the shape is ellipsoid, the others balling particles are round, balling particles and pores size is about 100 μm. The pores are interconnected to form a network. Additionally, the pore (size greater than 100 µm) shape of the sample surface was mostly meniscus-shaped [24]. If the scanning speed is too fast, due to the low density and chemical activity of magnesium, more powder is blown upwards and oxidized to form black fog (MgO), contaminating the mold cavity [29]. The relative density in this interval is 90.9–90.4%. In fact, the forming is in a solid-liquid state and there is poor bonding between the powder particles, so the sample has a powder accumulation structure without any mechanical strength at these processing parameters. It happens because the scanning speed is too fast (low energy input), the powder cannot be completely melted, resulting in the adjacent tracks cannot overlap, so the bonding neck between the powders is poor and large pores are formed, the relative density is low. It can also be seen from Figure 3b and Figure 4b that some ellipsoidal particles are distributed around the pores. In the SLM process, when the powder bed is irradiated by a high-energy laser beam, not only the powder in the center of the laser irradiation will instantly melt into a liquid molten pool, but the powder around the laser irradiation area will also be affected. Because the magnesium alloy powder is relatively light, the scanning line of the liquid molten pool can easily grasp the surrounding lightweight magnesium powder, and finally form a “glue powder phenomenon”. The ellipsoidal balling is caused by the following two reasons: first, the high scanning speed results in low energy density. On the one hand, the powder is not completely melted due to insufficient heating, so large-sized balling particles are formed. On the other hand, the wetting of the molten pool is poor because of the high scanning speed (low energy density), so the diffusion of the melt is blocked; second, the solidification rate increases at high scanning speeds. When liquid metal solidifies, it tends to form a spherical shape according to the principle of minimum free energy. Because of the fast solidification rate, the melt is completely solidified before it forms a spherical shape, so the ellipsoidal shape is retained. 

When the scanning speed decreases to 600–850 mm/s, it can be seen from the Figure 3e and Figure 4e,h that the balling particles and pores still exist on the surface. However, the size and number of pores and agglomerated balling particles decrease (Figure 3c) and the fusion between the powders improves (Figure 3f,g and Figure 4e–h). The energy density in this area is 55.1–78.1 J/mm^3^. Due to the decrease in scanning speed, the interaction time between powder and laser is prolonged, and the temperature of some particles is higher than the melting point, which causes the powder to partially melt. In this case, adjacent particles are sintered together due to the formation of a small amount of liquid [21]; however, the balling zone is still in a liquid-solid state. The relative density of magnesium alloy samples increases with the decrease of scanning speeds (i.e., energy input). Additionally, the relative density is 91.3–96.1%. At the same time, when the scanning speed is reduced to 650 mm/s, the surface of the sample began to show the scanning track line with the reduction of the scanning speed, which is represented by the red dotted line in Figure 4g–i. Between the two scanning tracks (dash line) is a “hill-like” protrusion. In the high magnification SEM image Figure 3g,h, it can be seen that the “hills” at this time still have the morphology of balling particles. The formation of hill topography is discussed in the next paragraph. The balling particles size is about 50 μm. The energy density increases when the scanning speed decreases, the temperature of the molten pool increases, so the powder is melted more fully after being irradiated by laser, and the wettability and molten pool fluidity are improved. 

Further reduce the scanning speed to 400–550 mm/s, the pores on the surface disappear, the amount of balling particles is further reduced, and the surface quality is relatively rough (Figure 3i–k). Based on the characteristics of layered manufacturing technology, the initially printed thin layer is in direct contact with the substrate and melts quickly. As the number of printed layers increases, the heat transferred from the substrate to the powder decreases, so the pores are larger. This phenomenon is due to the scanning speed being too fast (energy input is too low), resulting in incomplete powder melting and the formation of pores. However, as the scanning speed decreases and the energy density increases, the upper layer powder receives sufficient heat to melt the powder, which results in the improvement of the bonding neck between the powders and the disappearance of pores. Therefore, the relative density increased to 96.3–97.6%. Under the effect of decreased scanning speeds, the instantaneous temperature gradient from the center of the track to the edge increased, so the surface tension decreased, the viscosity of the metal droplets decreased, and the number of the balling particles reduced. It can also be seen from the low-magnification SEM (Figure 4i,j) that there are clear scanning track lines on the surface and marked with a red dotted line. Some “hills” are formed between the two scanning tracks, and these hills become smoother than the scanning speed at 600–850 mm/s, and the morphology of the balling particles is not obvious (Figure 3i and Figure 4i,j, red dotted line). The energy density in this area is 85.2–117.2 J/mm^3^. After the scanning speed is reduced, the energy density further increases, as a result, the temperature of molten pool increases, and the melt surface tension decreases. Since the scanning track is not a continuous melt, it is formed by discontinuous spherical particles, and the increase in energy density causes the spherical particles to be smoothed by surface tension. As the temperature of the molten pool increases, the melt is easier to diffuse due to the decrease in viscosity, so the shape of the “hill” is smoother than the scanning speed of 600–850 mm/s. However, the energy density is not enough to make the surface of the sample smooth.

As the scanning speed reduced to 250–350 mm/s, it is shows that the sample surface is gradually smooth and flat (Figure 3m–o), the “hills” disappear. The relative density is increased to 98.9–99.4%. Additionally, the energy density is 133.9–187.5 J/mm^3^. At this time, the scanning speed and energy density are more suitable, the powder is tightly bound, and the melt fusion performance is better. The temperature of the liquid phase was a certain level above the liquidus temperature. Then, the liquid phase rapidly diffused and solidified, and the melted powders were well combined to form a continuous and smooth trajectory [21]. With the decrease of scanning speed, the interaction time between powder and laser increases, and the heat that is received by powder per unit time increases. Therefore, the energy density in this area increases the temperature of the powder bed and reduces the viscosity of the molten pool, thus promoting the diffusion of the molten and the more effective densification of the solid powder particles. The relative density reached a maximum of 99.4% when the energy density is 156.2 J/mm^3^ (300 mm/s). Continue to reduce the scanning speed to 250 mm/s, the change of relative density is not obvious or even slightly reduced. This is because the energy input exceeds the optimal range, the temperature of the molten pool continues to rise, and the solid solution of Al element decreases due to the weakening of the solute capture effect, resulting in a slight decrease in the relative density [24]. The surface morphology and quality of the sample can be improved by adjusting the scanning speed, but the scanning speed should be controlled within the appropriate range. Too high scanning speed (low energy density) will cause the powder to be insufficiently heated and it cannot be completely melted. Too low scanning speed (high energy density) will affect the solute capture effect or cause the powder to evaporate. The best energy density in this study is 156.2 J/mm^3^ (the scan speed is 300 mm/s). The relative density of the sample is 99.4%. However, it is shown in Figure 4o that, even at a scanning speed of 250 mm/s (E_v_ is187.5 J/mm^3^), there are still a few small-sized balling particles on the surface. This is different from the result that the SEM image of the sample surface shows a smoother surface when the hatch spacing is 0.06 mm and the scanning speed is 350 mm/s (E_v_ is 178 J/mm^3^) in the previous work. In addition, the above results indicate that increasing the energy density to a suitable range is beneficial for reducing the balling. However, some studies have shown that when the energy density continues to increase beyond the appropriate range, the balling phenomenon will intensify [30,31]. The research has also shown that balling not only occurs at high scanning speeds, but also at low scanning speeds [31]. This indicates that the processing parameters can control the balling to a certain extent, but it has limitations. The control of balling requires further in-depth study.

The direct manifestation of the process parameters that deviate from the optimal conditions (parameter window) during the forming process is the balling phenomenon of the molten pool, which will directly lead to the increase of surface roughness, non-uniformity of subsequent powder coating, increase of defects, and other problems. Therefore, in order to control the final performance of the SLMed magnesium alloy, it is particularly important to find a method to control the balling, analyze the factors affecting the balling of the SLMed magnesium alloy, and seek the optimal temperature that is required to control the balling in the SLMed magnesium alloy forming process.

### 3.2. Effect of Wettability

The problem of balling is mainly related to the physical properties of the metal melt. In the SLM process, the powder is completely melted under the action of laser irradiation. The viscosity of the metal melt is small and the surface tension is large. It is easy to form a lot of scattered metal balls under the combined action of viscous flow and surface energy reduction.

**Figure 6:**
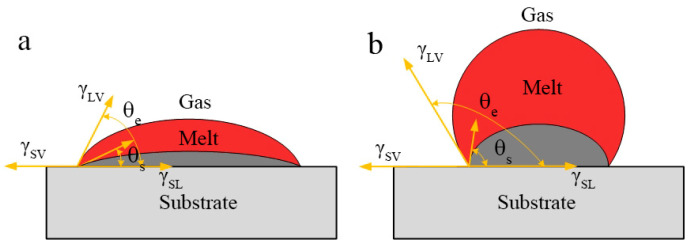
The schematic of liquid metal in the condition of: (**a**) wetting and (**b**) non-wetting.

In the SLM process, the expansion material needs to balance physical properties such as surface tension, melting point, and viscosity thermal conductivity. One of the causes of balling can be attributed to the wetting problem of liquid metal and solid surface. That is, when liquid drops onto a smooth and uniform solid surface, if it is not spread out, a liquid drop will be formed. Its shape is determined by the angle between the tangent line of gas-liquid interface made at the junction of solid-liquid interface and the solid-liquid interface, as shown in Figure 6. This angle is called the contact angle between the liquid and the solid surface, or wetting angle $\theta_{e}$.

If the molten pool wets itself after being irradiated by the laser, a simple SLM process model can be established, so-called Homologous Wetting [32]. Generally speaking, the process of SLMed magnesium alloy consists of two steps: first, thee powder is melted after receiving the heat from the laser; second, the melt is solidified on the substrate or the precursor layer. The second step is the homogenous wetting process. Homogenous wetting is the main mechanism of melt wetting on substrates of similar materials. This is a non-equilibrium process, including fluid flow, heat conduction, and solidification. According to the Young’s Equation (2),

(2) $$\gamma_{SV}-\gamma_{SL}=\gamma_{LV}\cos\theta_{e}$$

where $\gamma_{SV}$, $\gamma_{SL}$, and $\gamma_{LV}$ are the surface tension of the solid-gas, solid-liquid, and liquid-gas interface, respectively, $\theta_{e}$ is the contact angles. Combined with Figure 6, it can be seen that when the angle $\theta_{e}$ is 180° (cos $\theta_{e}$ = −1), it is completely non-wetting; if the $\theta_{e}$ is greater than 90° and less than 180° (−1 < cos $\theta_{e}$ < 0), the liquid easily shrinks into a spherical shape on the surface of the solid, that is, the liquid does not wet the solid (Figure 6a) and the energy density is corresponding to 49.3–117.2 J/mm^3^;On the contrary, if the $\theta_{e}$ is less than 90° (0 < cos $\theta_{e}$ *θ* < 1), the liquid is easy to spread on the solid surface, then the wettability is improved (Figure 6b) and the energy density is corresponding to 133.9–187.5 J/mm^3^; when the $\theta_{e}$ is 0° (cos $\theta_{e}$ = 1), it is completely wet, this is equivalent to the droplet spreading completely. In general, the smaller the angle $\theta_{e}$, the better the wettability. Therefore, in the SLM process, for the purpose of better forming surface quality, the surface tension of liquid-gas interface is usually changed by adjusting process parameters, so as to reduce the contact angle from 0° to 90° (0 < cos $\theta_{e}$ < 1), realize the continuous spreading of molten pool, improve the surface wetting ability of liquid metal, and, thus, improve the forming surface quality of parts. In order to obtain a smaller $\theta_{e}$, the cos $\theta_{e}$ should be close to 1, that is, the smaller the surface tension of the liquid-gas interface $\gamma_{LV}$, the closer the cos $\theta_{e}$ is to 1, the smaller the angle $\theta_{e}$, the better the liquid metal spreading performance of the molten pool. Therefore, the scanning speed is reduced, and the energy density is increased to an appropriate range, thereby improving the contact angle and surface tension, and the metal droplets are easy to spread, reducing the occurrence of balling. In addition, the viscosity of the melt changes with the adjustment of the processing parameters. Equation (3) shows the formula of dynamic viscosity *μ* [33],

(3) $$\mu =\frac{16}{15}\sqrt{\frac{m}{kT}}\gamma$$

where *γ*, *T*, *m*, and *k* represent the surface tension, temperature of the melt pool, atomic mass, and Boltzmann constant, respectively. When a slower scanning speed (larger energy density) is applied, the temperature of the molten pool increases, and the melt is more likely to diffuse smoothly around due to the decrease in surface tension and dynamic viscosity of the melt. Therefore, balling phenomenon is improved.

### 3.3. Effect of the Plateau-Rayleigh Capillary Instability

**Figure 7:**
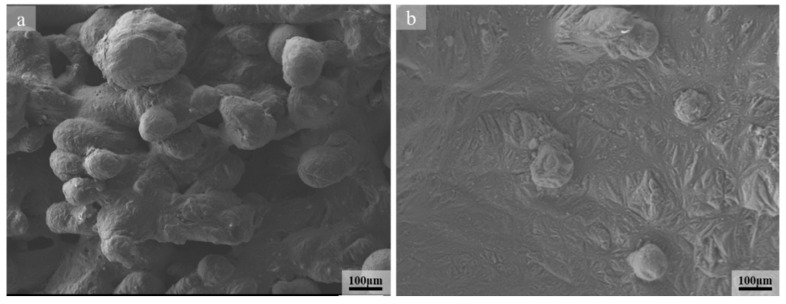
SEM images of SLM magnesium alloy under different energy densities: (**a**) is 26.79 J/mm^3^ and (**b**) 156.25 J/mm^3^.

For each track in the SLM process, due to the progressive scanning of the SLM process, the laser beam melts the powder particles to form a cylindrical melting trajectory. The instability of these liquid cylinders was first proposed by Rayleigh [34], which is called Plateau–Rayleigh capillary instability. Plateau–Rayleigh instability is expressed as a natural phenomenon in which liquid cylinders break into smaller melt droplets due to the surface tension. The Plateau–Rayleigh instability is due to the surface tension of the liquid tending to minimize the surface of the droplet. Therefore, Plateau–Rayleigh instability can be used to analyze the single-track forming process of the SLMed magnesium. As the feature size of the fluid decreases, the effect of interfacial tension becomes dominant, and the effect of Plateau–Rayleigh instability becomes more and more prominent. The stability of the liquid cylinder is related to the wavelength of the irradiated laser and the size of the cylinder, a sufficient and necessary condition for the continuous stability of a cylindrical liquid can be obtained Equation (4) [15,34],

(4) λ<πd

where *λ* represents the wavelength and *d* represents the initial diameter of the undisturbed cylinder. When this formula is satisfied, the liquid cylinder is stable under any disturbance. The larger the initial diameter of the cylinder, the longer it takes for the laser to break the cylinder to avoid agglomeration to form balling particles. That is, the more continuous the cylinder, the less likely it is to be broken into balling particles. When considering that the volume change of SLMed magnesium alloy cylinder during the crushing process is constant, so the size of the magnesium alloy liquid cylinder is positively related to the size of the agglomerate, and the diameter of the agglomerate generally increases with the energy input or decreases with the scanning speed [35], therefore reducing the scanning speed and increasing the energy density (Figure 3, Figure 4 and Figure 7) can prevent the liquid cylinder from being broken into agglomerates, thereby obtaining a continuous surface and reducing the balling rate [15]. 

Further, in order to be more in line with the molding characteristics of the SLM process, if a part of the liquid cylinder in a continuous single-track is in contact with the substrate, the optimization results of Plateau–Rayleigh capillary instability are shown in Equation (5) [36],

(5) $$\frac{\pi d}{\lambda }<\sqrt{2}\sqrt{\frac{\Phi (1+\cos2\Phi )-\sin2\Phi }{2\Phi (2+\cos\Phi )-3\sin2\Phi }}$$

For a single-track with contact angle Φ < Π/2, no matter how long the length of the liquid cylinder is, it is in a stable state. The experimental results show that the remelting zone can enhance the stability of single-track, so the degree of remelting between layers has a crucial role in reducing the effect of balling. Combined with the determination conditions of Plateau–Rayleigh liquid cylinder stability, it can guide the study of the optimal scan line length and processing parameters. Therefore, adjust the process parameters (reducing the scanning speed and increasing the energy density) to an appropriate range to form the optimal melting track length, thereby reducing balling. However, the above analysis is not enough to analyze the balling phenomenon when the energy density exceeds the appropriate range, so further discussion and verification of thermodynamics and kinetics are still needed.

### 3.4. Effects of Thermodynamic Factors

The influence of thermodynamics is also important for the control of balling. The molecules on the surface of the liquid metal have additional potential energy than the molecules in the liquid. This potential energy is only available when the molecule is on the surface, so it is called surface free energy, or surface energy for short. This potential energy puts an additional force on the liquid surface molecules, and the surface has a tendency to shrink to a minimum. This force is called surface tension. The powder undergoes a phase change process from solid to liquid after being heated and melted, and a free surface is formed. The action of the laser and the powder increases the powder surface energy after the molten pool is formed, and it is in an unstable state. Surface area is an important variable that affects the thermodynamic function of the system, according to Gibbs free energy properties. According to the principle of Gibbs minimum free energy, the system spontaneously moves in the direction of Gibbs free energy reduction. Because the spherical surface area is the smallest (that is, the surface energy is the smallest) at the same volume, the molten pool that is formed by SLM tends to spheroidize. As the surface area decreases, the reduction in surface energy is the main reason for the rupture of the liquid cylinder.

Under ideal circumstances, the surface tension at the center of the molten pool is small due to the high temperature, and the surface tension at the edge is high. The thermocapillary convection will flow from the center to the edge. This phenomenon is called Marangoni flow. However, the ideal Marangoni flow rarely exists. The energy of the SLM process is very high, so the temperature gradient between the center and the edge of the molten pool is large. Surface tension is related to temperature, so the surface tension at different locations in the molten pool is different due to the difference in temperature. The Marangoni flow affects the melting trajectory of the SLM sample and generates additional force, which affects the surface balling [37].

### 3.5. Effect of Dynamic Factors

The powder on the substrate will undergo a series of processes of melting, wetting, spreading, and solidification as the laser spot moves. In fact, the spreading behavior of SLM droplets is very complicated: on the one hand, the capillary driving force competes with inertial resistance; on the other hand, the spreading process competes with the rapid solidification process under high temperature gradients. In such a competitive process, once the spreading is blocked, it will cause the droplets to spheroidize. 

The spreading and solidification process are carried out at the same time. Balling depends on whether the droplet spreads quickly or solidifies quickly under wetting conditions. Therefore, the processing of spreading and solidification of magnesium alloy metal droplets are analyzed and discussed separately. Additionally, the model of spreading and solidification are established basic on the result of experiment.

If the solidification process is dominant, the spreading process is slow, which easily leads to balling. Conversely, if the spreading process is dominant, the solidification process is slow, so the metal melt is completely spread before being solidified, inhibiting the occurrence of balling. It is worthwhile to establish a solidification and spreading model.

#### 3.5.1. The Spreading Dynamics of Metal Droplet

Firstly, the process of droplet spreading is analyzed without considering the influence of temperature gradient. The droplet spreading process is affected by two dimensionless parameters, the Weber number and the Ohnesorge number [24]. The Weber number measures the driving force of the droplet diffusion on the substrate, which is, the driving force for spreading, and the Ohnesorge number is the resistance to diffusion, which is, the resistance to the spreading process. Among them, the Weber number (W_e_) is expressed by Equation (6), and the Ohnesorge number (O_h_) is expressed by Equation (7) [38],

(6) $$W_{e}=\frac{\rho_{e}\cdot V^{2}\cdot r}{\sigma }$$

(7) $$O_{h}=\frac{\mu }{(\rho_{e}\cdot \sigma \cdot r{)}^{1/2}}$$

where *r*, *V*, *ρ*_e_, *μ*, and *σ* represent the radius of spherical droplet, droplet impact velocity, melt density, viscosity, and surface tension, respectively. According to the SEM image, and for the convenience of calculation, the typical droplet size is taken to be 100 μm. Not only the Mg is considered, but also several common metals used in SLM are introduced for comparison, such as Al, Fe, Cu, Ni, Ti, in order to more comprehensively analyze the spreading and solidification process of metal droplets. Table 3 shows the physical properties of different metals. It can be known from the calculation of physical parameters of magnesium that the O_h_ = 0.0042 (O_h_ << 1), and the *V* of the droplet tends to 0 during SLM process, so W_e_ << 1, the calculation results of the other metals also show the same trend. According to the calculation results, both W_e_ and O_h_ are relatively small, which indicates that the spreading process of SLM droplets is driven by capillarity, and the forces that hinder spreading are mainly inertial forces (not viscosity), and the metal droplets spread and solidify under the competition of capillarity and inertial force [38]. 

The spreading and solidification of metal droplets actually restrict each other. If the time that is required for the droplets to spread completely is lower than the solidification time, it means that the droplets spread before the solidification, and the phenomenon of balling can be avoided. Conversely, if the time required for full spreading is longer than the solidification time, the metal liquid will not spread completely when solidified, causing balling. Therefore, it is necessary to determine the spreading time and solidification time of the metal droplets, so as to judge the key factors to control the balling.

It is assumed that the spreading and solidification of metal droplets on the substrate occur under isothermal conditions in order to simplify the calculation, without considering the temperature gradient. If the liquid diffuses to a moderate contact angle, (not too close to zero or to a Π angle), the characteristic diffusion velocity *v* and characteristic diffusion time *t* of the droplet have a specific form, as shown in Equations (8) and (9) [38],

(8) $$t=(\frac{\rho_{e}r^{3}}{\sigma }{)}^{1/2}$$

(9) $$\nu =(\frac{\sigma }{\rho_{e}r}{)}^{1/2}$$

Table 4 shows the spreading time and speed of different metal droplets. When compared with other metals, the spreading time of Mg under isothermal conditions is 53.3 μs, the spreading speed is 1.86 m/s, the spreading speed is faster, and the time required for full spreading is shorter. When comparing several metals, Cu has the longest spreading time and the lowest spreading speed, Ti has the shortest spreading time and the fastest spreading speed, the order of spreading time is Cu > Ni > Fe > Mg > Al > Ti, indicating that magnesium is easy to spread during the SLM process, the balling rate is lower than other metals, and it has better formability. However, in the actual production process, balling of SLM magnesium still exists and it is quite serious. At this time, it is required to comprehensively consider the spreading and solidification process to obtain the balling control mechanism. For isothermal deposition, the universal droplet spreading equation can be expressed as Equation (10) [38],

(10) $$\frac{R}{r}=2.4[1-\exp(-0.9t/\sqrt{\rho a^{3}/\sigma })]$$

where *R* is the contact radius between the droplet and the substrate and $t/\sqrt{\rho a^{3}/\sigma }$ is the dimensionless time (less than 3).

#### 3.5.2. The Solidification Kinetics of Metal Droplet

In practical applications, a more complex situation needs to take into account. In addition to analyzing the spread of the droplet, it is also necessary to analyze the solidification behavior of the droplet. Therefore, the balling behavior of SLMed magnesium alloy can be interpreted by establishing a competitive model of droplet diffusion and solidification. In this competitive mode, there are two simultaneous processes: After the magnesium alloy powder is irradiated by laser, the powder laid on the substrate is heated and melted (100 μm molten droplet), and the spreading process begins (with the reduction of the $\theta_{a}$), which forms a dynamic contact angle with the substrate $\theta_{a}$.The solidification process also takes place at the same time (with the increase of solidification angle $\theta_{s}$), $\theta_{s}$ represents the contact angle between the solidified part and the substrate, the two processes of spreading and solidification compete with each other.

For a given material, such as magnesium, this dynamic contact angle mainly depends on the Stefan number based on the temperature gradient between the melting point (solidus temperature) and the temperature of the solid covered by the melt, as shown in the formula $S=C(T_{f}-T_{t})/L$. *C*, *L*, $T_{f}$, and $T_{t}$ represent the specific heat capacity of the material, the latent heat of phase change, the solidus temperature and substrate temperature, respectively. The droplet spreading can only occur under the condition of $\theta_{a}>\theta_{s}$. When the dynamic contact angle $\theta_{a}$ controlled by the spreading speed approaches the solidification angle $\theta_{s}$ controlled by the temperature gradient, the bottom of the droplet is completely solidified and the capillary driving force is lost. At this time, the droplet no longer has the possibility of continued spreading. When the angle $\theta_{a}$ approaches the angle $\theta_{s}$ infinitely, the dynamic contact angle of the solidification front is represented by $\theta_{*}$. The function *F*(*S*) can be expressed as $F(S)\approx \theta_{*}$. It can be seen that it is not only related to the characteristics of the material, but also lies on the thermal conditions of $T_{f}\;-T_{t}$. Changing the substrate temperature can also play a role in suppressing balling. 

It is far from enough to determine the spreading time of magnesium droplets alone, and it needs to be measured from the aspects of spreading time and solidification time in order to fully explore the balling behavior of magnesium alloy droplets. The solidification process of magnesium droplet is analyzed and compared with other metals. The complete solidification time *t_s_* of the droplet under one-dimensional heat dissipation condition can be expressed as Equation (11) [40],

(11) $$t_{s}=2(\frac{r^{2}}{3D})\ln(\frac{T_{0}-T_{b}}{T_{s}-T_{b}})$$

*D* represents the diffusion coefficient, *T*_0_ represents the metal droplet temperature, *T_b_* is the substrate temperature (288 K), and *T_s_* is the solidus temperature. The solidification process is the process of decreasing from *T*_0_ to *T_b_*, and the time that is required for solidification is the solidification time. Because of the difference in thermodynamic data in different literatures, the data in literature, books, and JMatPro software (around melting point) are used for calculation, and then comprehensive analysis [41,42,43,44,45,46,47,48]. 

**Figure 8:**
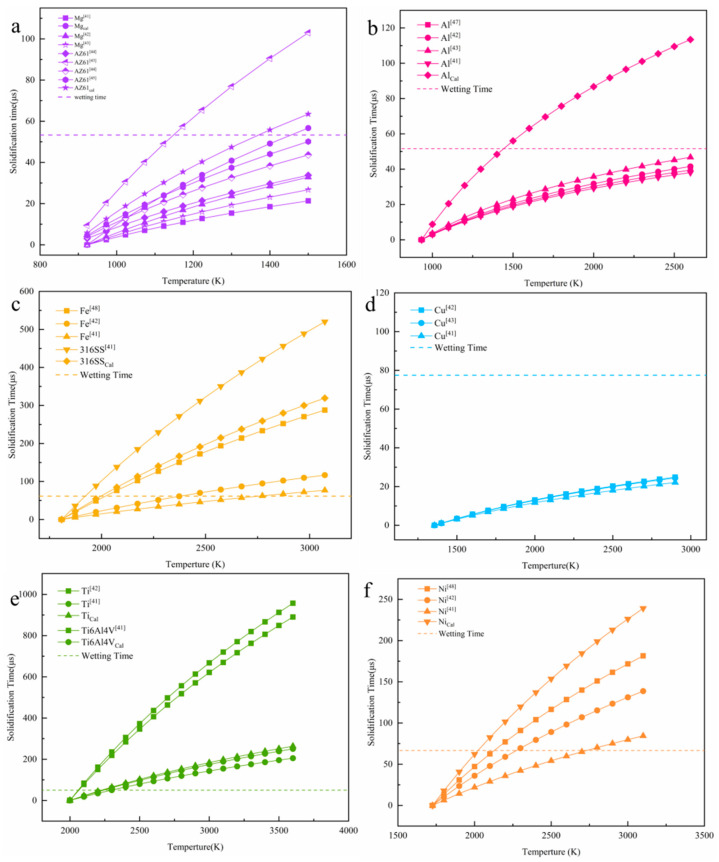
Calculation relationship between metal droplet temperature and solidification time of different metal melts (**a**) Mg; (**b**) Al; (**c**) Fe; (**d**) Cu; (**e**) Ti; and, (**f**) Ni. The dotted line in the Figure represents the calculated spreading time.

**Figure 9:**
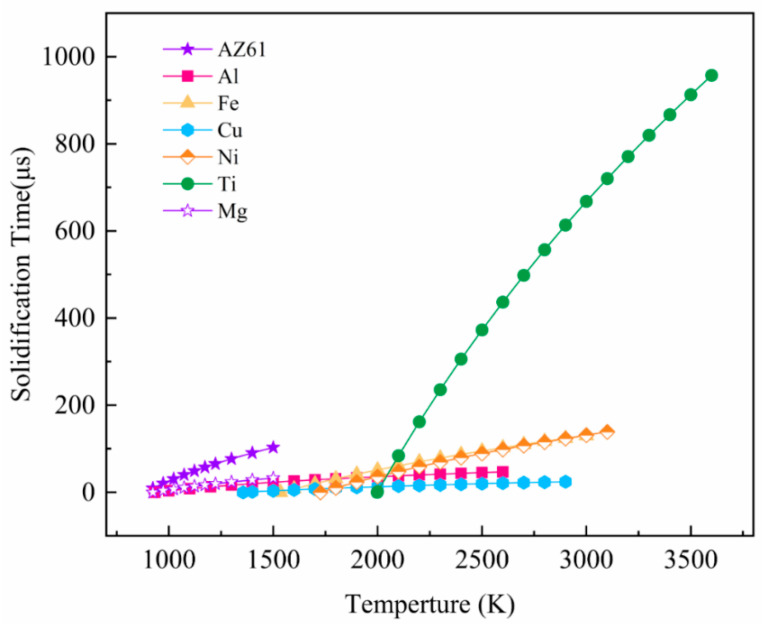
Comparison of different metal melt solidification processes.

Figure 8 calculates the relationship between different metal melt spreading and solidification according to the model. The solid line in the figure represents the time required for the solidification of the metal melt at different temperatures, and the dashed line indicates the time required for complete spreading. Figure 9 compares the typical solidification time-temperature curves of different metals, which can more intuitively compare the solidification behavior of different metals. If the spreading time is longer than the solidification time, it means that the material droplets are solidified before being fully spread, the trend of balling is obvious; If the spreading time is faster than the solidification time, it means that the material droplet spreading process is faster, and it is solidified after it is completely spread, which is not easy to spheroidize in the forming process.

It can be seen from the curve (Figure 9) that the slope from large to small is: Ti > Ni > Fe > AZ61 > Mg, Al. Fe, Ni, and Ti significantly prolong the solidification time as the melt temperature increases. The melt temperature has little effect on the solidification time of Cu. The solidification time of the melt cannot be significantly extended, even if the temperature is increased. However, the solidification time of Mg and Al metal melts will increase with increasing temperature, and the balling phenomenon will weaken. It shows that balling can be controlled by adjusting appropriate process parameters and energy input. Combined with Figure 8 and Figure 9, these metals can be classified into three categories according to the relationship between solidification time and spreading time. The first type is Ti, Fe, and Ni (Figure 8c–f), the balling of these three metals is easy to control. As the melt temperature increases, the solidification time of the droplets increases rapidly (the slope is larger). When the melt temperature is around 2100–2300 K, the solidification time of the three metals has exceeded the spreading time. This shows that, within a suitable parameter window, the temperature of the droplet is higher, and its spreading process is dominant, so that it can avoid balling and ensure the forming performance. The second type is Cu (Figure 8d). Theoretical calculations indicate that the balling of SLMed Cu is difficult to control (the slope of the curve is small). As the melt temperature increases, the solidification time does not increase significantly. Even if the temperature reaches near the boiling point, the spreading time is still long, and the solidification process is dominant, which results in balling of the Cu droplets. It shows that copper has a severe tendency to spheroidize during the SLM process, and its quality is difficult to control. The third type is Mg and Al (Figure 8a,b), which are two special metals. The slope of the curve of these two metals is small, however, the melt density of the two metals is also small, and so the spreading speed is very fast. The solidification time of pure Mg melt is 18–49 μs near the boiling point, which is still lower than the spreading time, indicating that the balling of pure Mg droplets is difficult to control. However, as the temperature of pure Mg droplets increases, the solidification time increases. 

It can be seen from the Figure 8a that the solidification time of AZ61 is 57.5 μs near 900 °C (1173 K), which is longer than the wetting time (53.3 μs), so the spreading process is dominant. The optimal temperature for SLMed AZ61 magnesium alloy is obtained through the theoretically calculated. At this temperature, balling can be minimized and dense forming can be achieved. This can also explain the balling behavior under the deviation from the optimal parameter window. If the scanning speed is too slow and the energy density is too high, at this time, although the temperature of the droplets delays the solidification process, the increase of the droplet volume slows down the spreading process, and the solidification process is dominant and causes balling. If the scanning speed is too fast and the heat input is low, the temperature of the droplet drops, the solidification process is dominant, and balling occurs. The dynamic spreading of molten pool, the analysis of wetting and solidification process, and the establishment of SLM balling model can provide reference for the design of SLM forming parameters of Mg and other different metals. The change of solidification time of Mg metal droplets with temperature and the optimal temperature can provide a reference for controlling balling.

### 3.6. Outlook

This work exposes the balling mechanism of the SLMed Magnesium alloy and analyzed the balling phenomenon of Mg in the laser processing from the aspects of Mg inherent metal properties and laser processing. From the laser processing point of view, the best energy density is 133.9–187.5 J/mm^3^. In terms of the inherent properties of the metal, the Mg droplet temperature needs to be controlled at 1173 K (900 °C) due to the current limitations of experimental conditions. However, it is difficult to accurately measure the actual temperature of the droplet corresponding to the optimal energy density, and experiments at high energy density (higher than 187.5 J/mm^3^) are limited due to severe evaporation, which hinders the verification of theory and experiments. However, this work can provide a theoretical and practical reference for the forming process of SLMed Mg and other metals. In the next step, the surface balling of SLMed magnesium alloy under higher energy input (higher than 187.5 J/mm^3^) should be further discussed to complete the experimental verification and the relationship between the actual temperature of the metal droplet and the corresponding energy density still needs further study. In addition, the kind and type of laser will affect the microstructure and mechanical properties of SLM samples. Firstly, in terms of laser type, the selection of continuous wave or pulse wave will directly affect the size and shape of the grain. It was reported that the microstructure in the center of the laser melting track is fine and uniform under the continuous wave laser irradiation. When compared with continuous wave radiation, the microstructure under pulsed radiation is composed of incompletely grown grains [49]. This is because the peak temperature of the melting layer is higher than the recrystallization temperature after being irradiated by the laser. The laser irradiation time in the continuous wave mode is longer than that in the pulse mode, and complete recrystallization and complete grain growth appear in the melting zone. Moreover, the solidification rate under pulsed laser irradiation is higher than that under continuous wave irradiation, which also inhibits the full growth of grains [50,51]. The difference in microstructure under the two laser modes will affect the mechanical properties of SLMed samples. In terms of kinds of laser, magnesium alloys have different absorption rates for different lasers, so they have different effects on SLM forming. It was reported that, as compared to CO_2_ lasers, due to the shorter wavelength of Nd:YAG lasers, the threshold irradiance that is required to melt the powder at the same penetration depth and scanning speed is reduced, so it is significantly better than CO_2_ lasers [52]. It was also reported that the high peak power of pulsed Nd:YAG laser would be more effective than the CW CO_2_ laser to disperse the surface oxide layer [4,53]. However, magnesium oxide (MgO) has an absorption rate of ~20% for Nd:YAG laser beams and an absorption rate of 93–98% for CO_2_ laser beams [52]. Therefore, the influence of the two kinds of lasers on the oxide layer on the surface of SLMed magnesium alloy and the morphology and performance of SLMed magnesium alloy under different types of laser modes are interesting issues that are worthy of study in the next step.

## 4. Conclusions

In this work, the effect of processing parameters, energy input and material properties on the macroscopic surface morphology when the scanning speeds from 250 to 950 mm/s and hatch spacing is 0.08 mm. Balling mechanism of SLMed AZ61 magnesium alloy has also been investigated and some conclusions have been obtained.

(1) In the direction of increasing scanning speed, the energy density decreases, and the phenomenon of balling and porosity on the sample surface deteriorated. This is mainly attributed to the lack of energy input, resulting in a lower molten pool temperature, an increase in liquid surface tension and viscosity, and the liquid cannot smoothly flow and migrate to around. The optimal energy density range is 133.9–187.5 J/mm^3^, the relative density of the magnesium alloy sample is between 98.9–99.4%. The best energy density is 156.2 J/mm^3^ (scanning speed is 300 mm/s), and the relative density of the sample is 99.4%.
(2) The driving force of the spreading behavior of SLMed magnesium alloy droplets is capillary force, and the spreading resistance is inertial force, which is related to the material itself characteristics, such as droplet size, viscosity, and surface tension. The solidification behavior of the droplets involves with the temperature. Affected by the temperature gradient, the specific heat capacity, the thermal conductivity, and the temperature difference between the melt and the substrate will change accordingly. The spreading and the solidification process of magnesium droplets are carried out simultaneously and compete with each other.
(3) The Mg droplet spreading/solidification kinetic model during the balling process has been established. The results show that the viscosity and surface tension of the magnesium melt are small, and the spreading process is fast. Although the solidification time of Mg melts changes slowly with the increase of temperature, the spreading speed is very fast due to the low melt density. Theoretically calculated that when the AZ61 melt is near 1173 K (900 °C), the spreading process is dominant, which can minimize the balling and realize the densification of SLMed Mg.

## Figures and Tables

**Table 1:** Chemical composition of AZ61.

Elements	Mg	Al	Zn	Mn	Si	Fe	Cu	Ni	O
**wt.%**	balance	6.3	1.2	0.3	0.06	0.03	0.01	0.01	0.087

**Table 2:** SLM processing parameters.

Processing Parameters	Value
Laser power, P	150 W
Scanning speed, v	250–950 mm/s
Laser beam spot size, D	70 μm
Hatch spacing, H	80 μm
Powder layer thickness, h	40 μm
Powder size distribution	30–70 μm

**Table 3:** Physical properties of metal droplets [39].

Metal	Melting Point *T*_e_/K	Melt Density *ρ*_e_/(kg·m^−3^)	Surface Tension *σ*/(mN·m^−1^)	Viscosity *μ*/(mPa·s)	O_h_
Mg	923	1590	559	1.25	0.00419
Al	933	2380	914	1.30	0.00278
Fe	1808	7030	1862	6.92	0.00604
Cu	1356	8000	1330	4.00	0.00388
Ni	1726	7900	1778	5.5	0.00464
Ti	1998	4130	1650	5	0.00606

**Table 4:** The estimated spreading characteristics of melt droplets.

Metal	Spreading Time, *t*/(μs)	Spreading Velocity, *v*/(m·s^−1^)
Mg	53.3	1.88
Al	51.6	1.96
Fe	61.4	1.62
Cu	77.5	1.29
Ni	66.7	1.50
Ti	50.0	1.99
